# Supplementary material for: Red-Brown Pigmentation of Acidipropionibacterium jensenii Is Tied to Haemolytic Activity and cyl-Like Gene Cluster
Source: Microorganisms. 2019 Oct 30;7(11):512. doi: 10.3390/microorganisms7110512 (PMC6920887; doi:10.3390/microorganisms7110512)
Supplement: Supplementary file 1 [file microorganisms-07-00512-s001.zip › Figure S1.pdf]

Acidipropionibacterium jensenii JS279

Created 08/15/19

(CP025571)

Chromosome (CP025571)

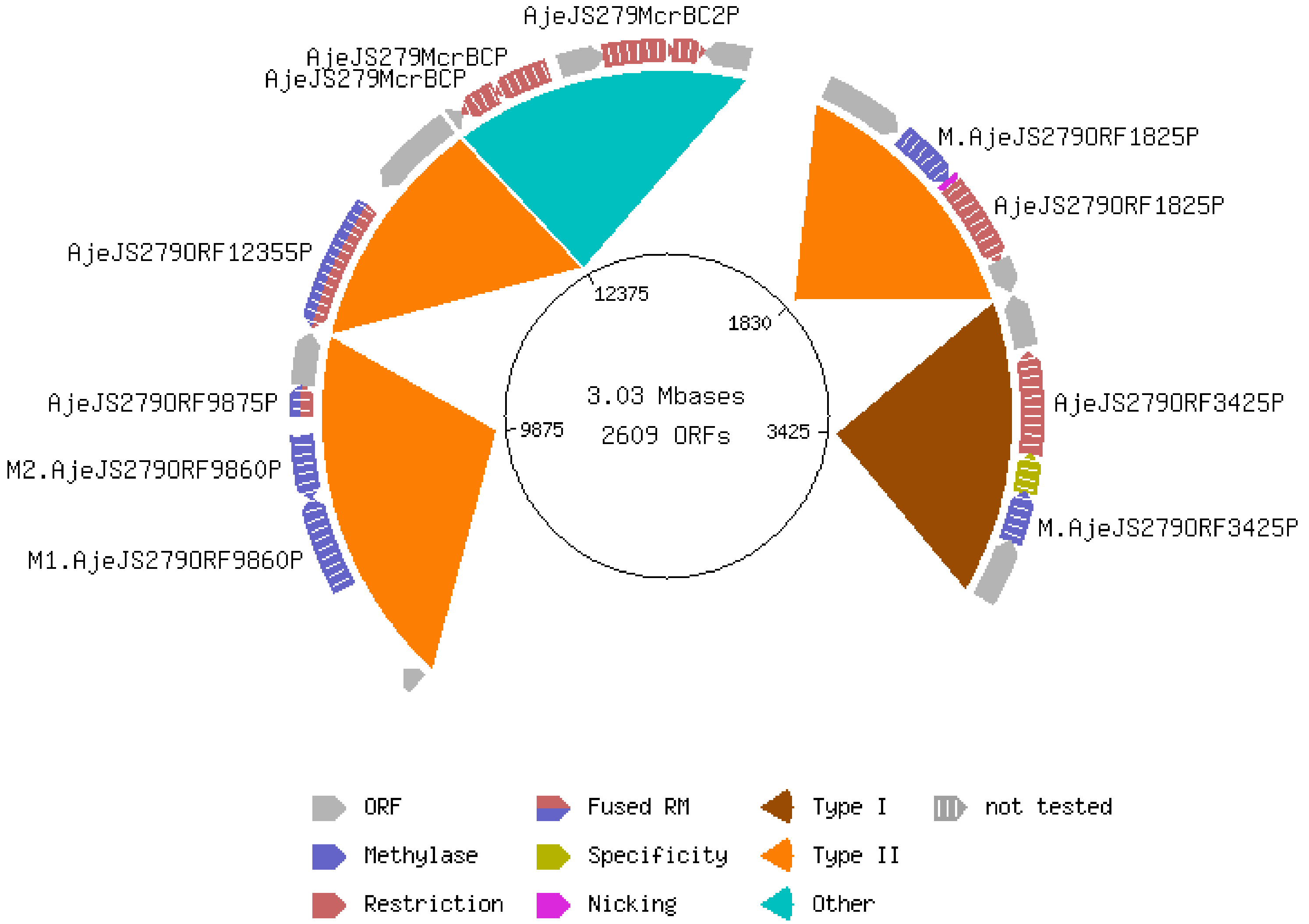

Type I

AjeJS279ORF3425

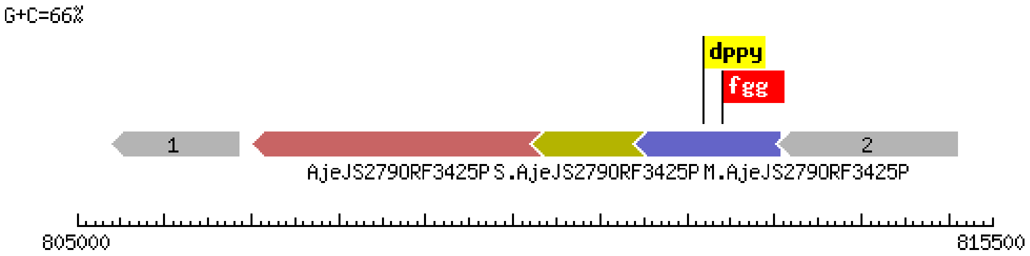

1	805411..806841	C0Z11_03410 glutamate decarboxylase
AjeJS279ORF3425P	807030..810365	--
S.AjeJS279ORF3425P	810230..811474	--
M.AjeJS279ORF3425P	811426..813060	C0Z11_03425 SAM-dependent DNA methyltransferase
2	813060..815105	C0Z11_03430 hypothetical protein

Type II

AjeJS279ORF1825

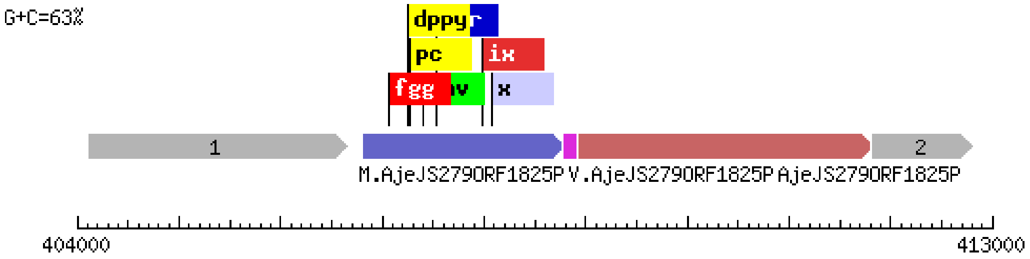

1	404116..406647	C0Z11_01820 ATP-dependent Clp protease ATP-binding subunit
M.AjeJS279ORF1825P	406827..408782	--
V.AjeJS279ORF1825P	408791..409207	C0Z11_01830 very short patch repair endonuclease
AjeJS279ORF1825P	408936..411836	--
2	411833..412798	C0Z11_01840 hypothetical protein

AjeJS279ORF9860

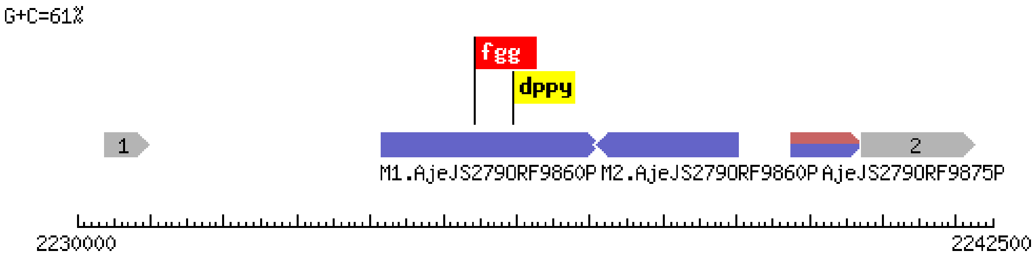

1	2230375..2230974	C0Z11_09850 DUF1788 domain-containing protein
M1.AjeJS279ORF9860P	2234147..2237116	--
M2.AjeJS279ORF9860P	2237094..2239019	--
AjeJS279ORF9875P	2239746..2240705	C0Z11_09875 class I SAM-dependent DNA methyltransferase
2	2240716..2242239	C0Z11_09880 AAA family ATPase

AjeJS279ORF9875

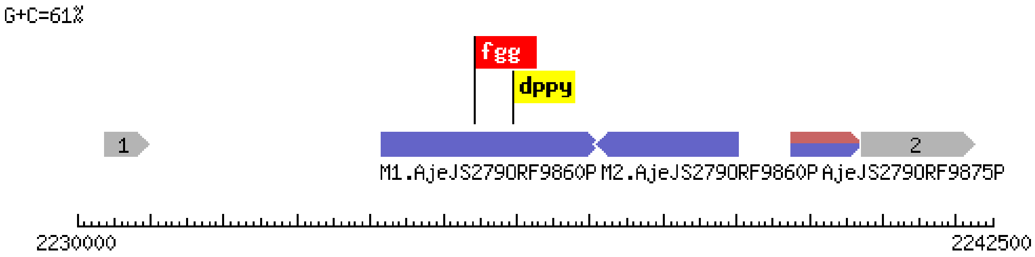

1	2230375..2230974	C0Z11_09850 DUF1788 domain-containing protein
M1.AjeJS279ORF9860P	2234147..2237116	--
M2.AjeJS279ORF9860P	2237094..2239019	--
AjeJS279ORF9875P	2239746..2240705	C0Z11_09875 class I SAM-dependent DNA methyltransferase
2	2240716..2242239	C0Z11_09880 AAA family ATPase

AjeJS279ORF12355

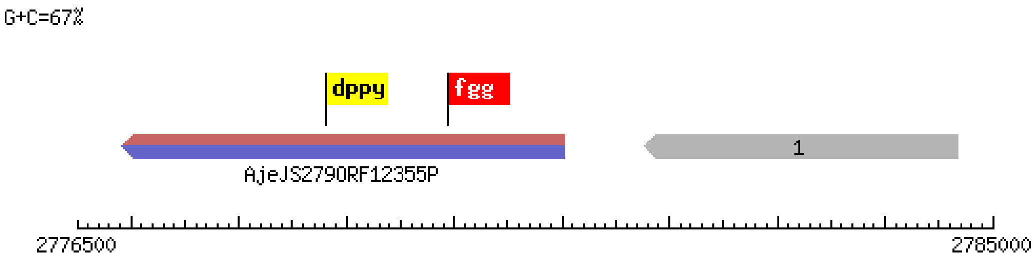

AjeJS279ORF12355P	2776921..2781015	--
1	2781766..2784675	C0Z11_12365 ATP-dependent helicase

Type IV

AjeJS279McrBC

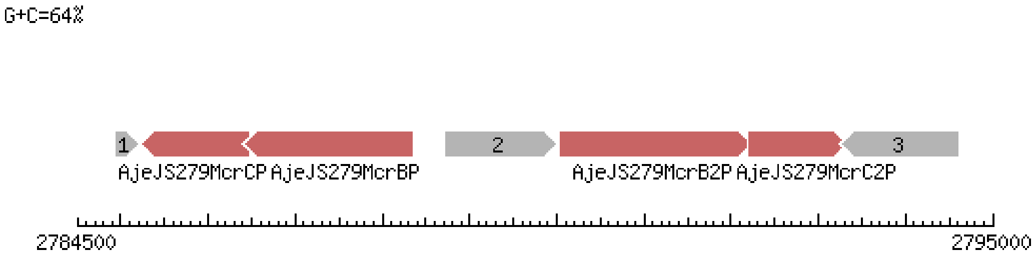

1	2784946..2785188	C0Z11_12370 hypothetical protein
AjeJS279McrCP	2785262..2786449	C0Z11_12375 restriction endonuclease
AjeJS279McrBP	2786424..2788343	C0Z11_12380 DUF4357 domain-containing protein
2	2788729..2789979	C0Z11_12385 hypothetical protein
AjeJS279McrB2P	2790036..2792207	C0Z11_12390 restriction endonuclease
AjeJS279McrC2P	2792204..2793295	C0Z11_12395 5-methylcytosine-specific restriction
3	2793283..2794590	C0Z11_12400 DUF1343 domain-containing protein

AjeJS279McrBC2

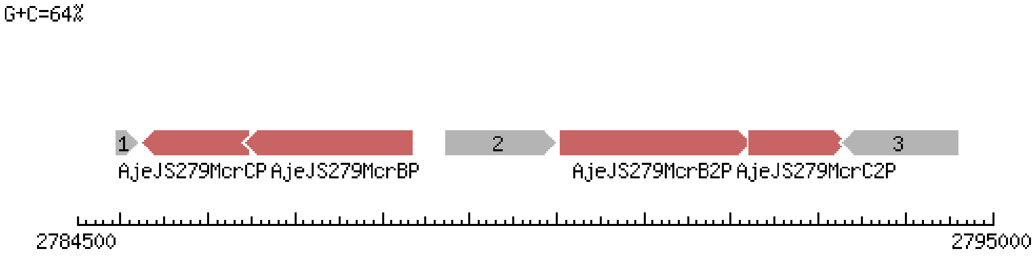

1	2784946..2785188	C0Z11_12370 hypothetical protein
AjeJS279McrCP	2785262..2786449	C0Z11_12375 restriction endonuclease
AjeJS279McrBP	2786424..2788343	C0Z11_12380 DUF4357 domain-containing protein
2	2788729..2789979	C0Z11_12385 hypothetical protein
AjeJS279McrB2P	2790036..2792207	C0Z11_12390 restriction endonuclease
AjeJS279McrC2P	2792204..2793295	C0Z11_12395 5-methylcytosine-specific restriction
3	2793283..2794590	C0Z11_12400 DUF1343 domain-containing protein
