# Supplementary material for: Red-Brown Pigmentation of Acidipropionibacterium jensenii Is Tied to Haemolytic Activity and cyl-Like Gene Cluster
Source: Microorganisms. 2019 Oct 30;7(11):512. doi: 10.3390/microorganisms7110512 (PMC6920887; doi:10.3390/microorganisms7110512)
Supplement: Supplementary file 1 [file microorganisms-07-00512-s001.zip › Figure S2.pdf]

Acidipropionibacterium jensenii JS280

Created 08/15/19

(CP025570)

Chromosome (CP025570)

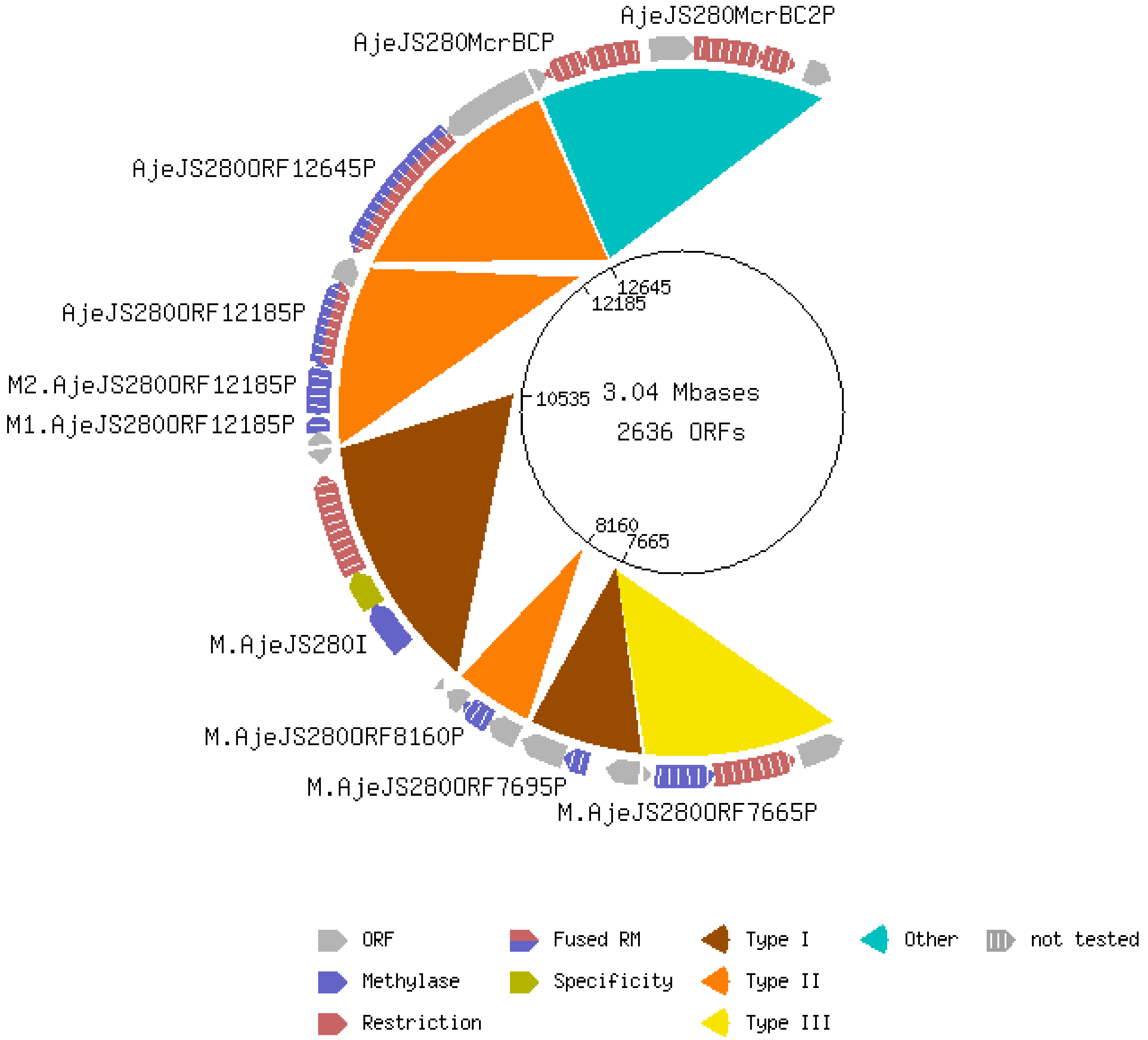

Type I

AjeJS280ORF7695

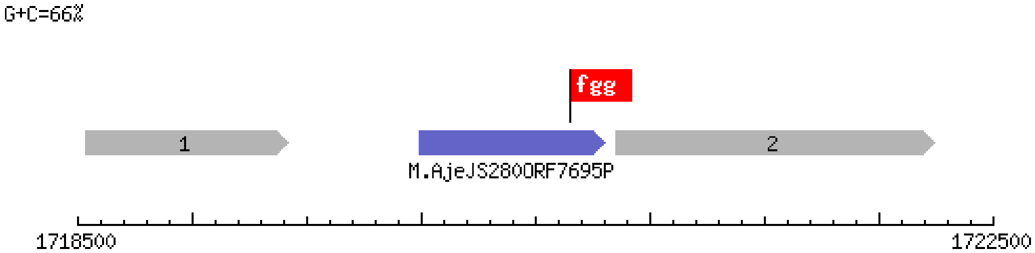

1	1718533..1719417	C0Z10_07690	IS30-like element ISPfr9 family transposase
M.AjeJS280ORF7695P	1719997..1720806	C0Z10_07695	SAM-dependent DNA methyltransferase
2	1720854..1722242	C0Z10_07700	IS1380 family transposase

AjeJS280I

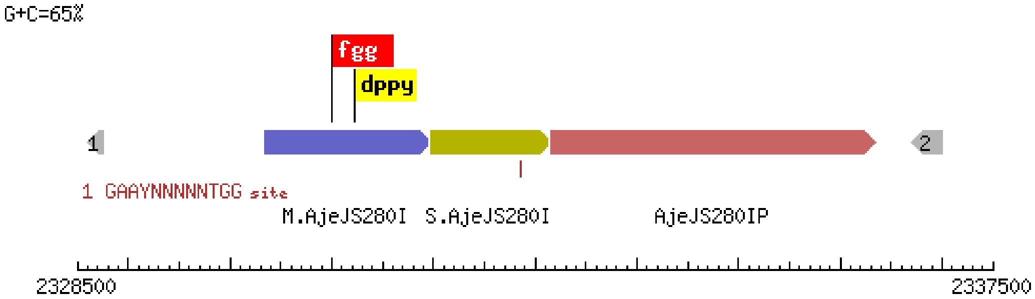

1	2328599..2328748	C0Z10_10530	3-isopropylmalate dehydrogenase
M.AjeJS280I	2330340..2331971	C0Z10_10535	SAM-dependent DNA methyltransferase
S.AjeJS280I	2331971..2333152	C0Z10_10540	restriction endonuclease subunit S
AjeJS280IP	2333152..2336337	C0Z10_10545	type I restriction endonuclease subunit R
2	2336709..2337008	C0Z10_10550	hypothetical protein

Type II

AjeJS280ORF8160

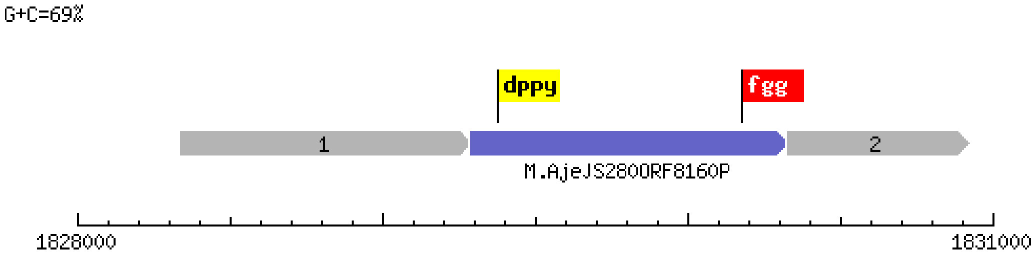

1	1828341..1829291	C0Z10_08155	hypothetical protein
M.AjeJS280ORF8160P	1829288..1830328	C0Z10_08160	site-specific DNA-methyltransferase
2	1830325..1830918	C0Z10_08165	antirestriction protein ArdA

AjeJS280ORF12185

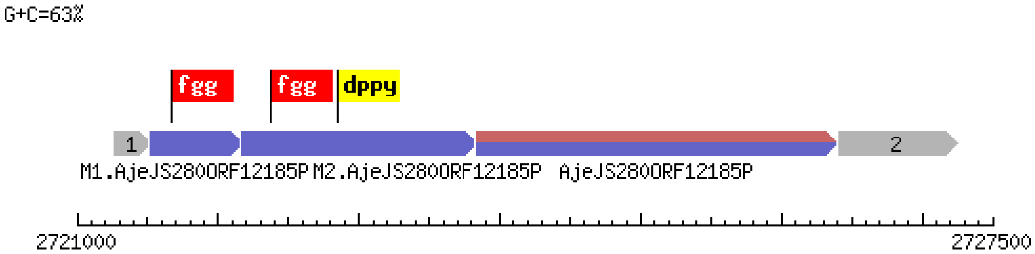

1	2721268..2721522	C0Z10_12180	helix-turn-helix domain-containing protein
M1.AjeJS280ORF12185P	2721519..2722172	C0Z10_12185	restriction endonuclease subunit M
M2.AjeJS280ORF12185P	2722169..2723842	C0Z10_12190	restriction endonuclease
AjeJS280ORF12185P	2723839..2726400	C0Z10_12195	restriction endonuclease
2	2726406..2727242	C0Z10_12200	hypothetical protein

AjeJS280ORF12645

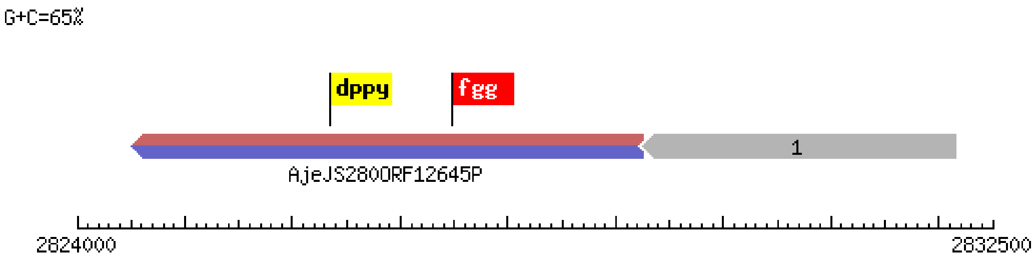

AjeJS280ORF12645P	2824499..2829256	C0Z10_12645 restriction endonuclease subunit M
1	2829256..2832153	C0Z10_12650 ATP-dependent helicase

Type III

AjeJS280ORF7665

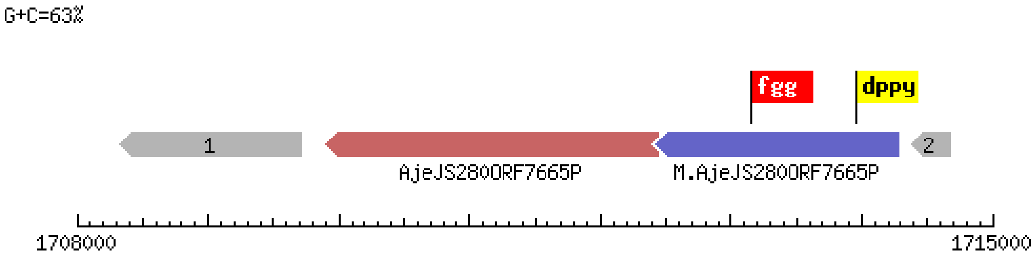

1	1708328..1709716	C0Z10_07655 IS1380 family transposase
AjeJS280ORF7665P	1709909..1712434	C0Z10_07660 restriction endonuclease
M.AjeJS280ORF7665P	1712431..1714281	C0Z10_07665 site-specific DNA-methyltransferase
2	1714378..1714665	C0Z10_07670 hypothetical protein

Type IV

AjeJS280McrBC

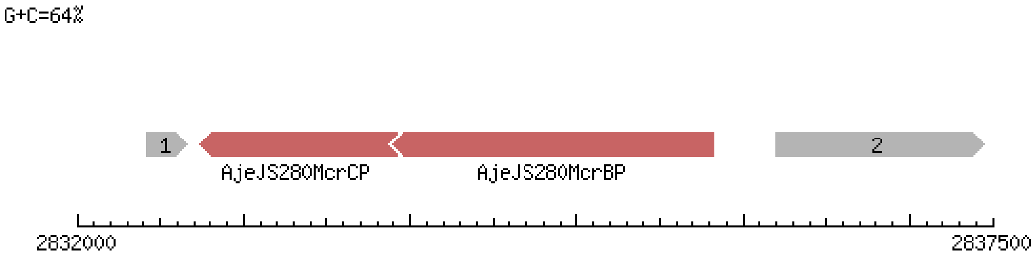

1	2832420..2832662	C0Z10_12655 hypothetical protein
AjeJS280McrCP	2832735..2833922	C0Z10_12660 restriction endonuclease
AjeJS280McrBP	2833897..2835816	C0Z10_12665 DUF4357 domain-containing protein
2	2836197..2837447	C0Z10_12670 hypothetical protein

AjeJS280McrBC2

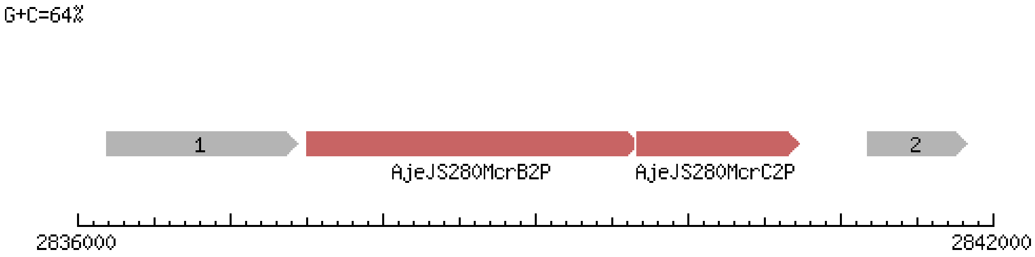

1	2836197..2837447	C0Z10_12670 hypothetical protein
AjeJS280McrB2P	2837504..2839675	C0Z10_12675 restriction endonuclease
AjeJS280McrC2P	2839672..2840724	C0Z10_12680 5-methylcytosine-specific restriction
2	2841177..2841836	C0Z10_12685 biotin transporter BioY
