# Supplementary material for: Red-Brown Pigmentation of Acidipropionibacterium jensenii Is Tied to Haemolytic Activity and cyl-Like Gene Cluster
Source: Microorganisms. 2019 Oct 30;7(11):512. doi: 10.3390/microorganisms7110512 (PMC6920887; doi:10.3390/microorganisms7110512)

Figure S3. Progressive Mauve alignment of genomes reveals considerable differences in genome organization between strains due to large inversions.

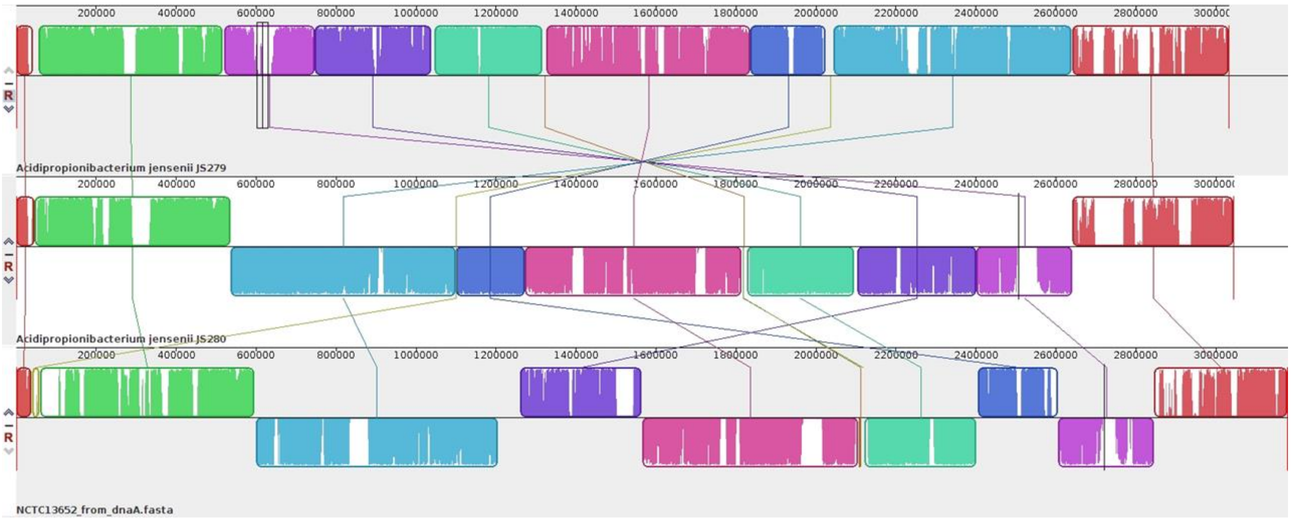

Supplement: Supplementary file 1 [file microorganisms-07-00512-s001.zip › Figure S3.pdf]
