# Supplementary material for: Red-Brown Pigmentation of Acidipropionibacterium jensenii Is Tied to Haemolytic Activity and cyl-Like Gene Cluster
Source: Microorganisms. 2019 Oct 30;7(11):512. doi: 10.3390/microorganisms7110512 (PMC6920887; doi:10.3390/microorganisms7110512)

Figure S4 Venn diagram representing distribution of the total of 2599 orthologous groups of genes in strains JS279, JS280 and *A. jensenii* NCTC13652 (equivalent to type strain DSM 20535) as predicted by ROARY.

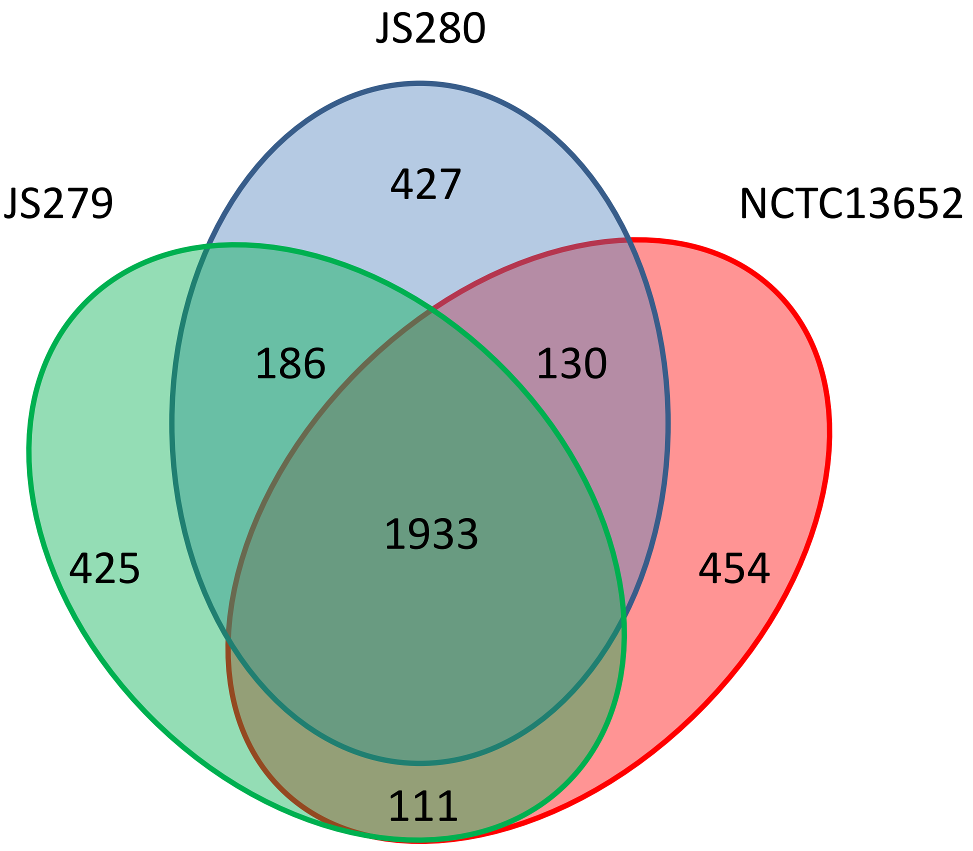

Supplement: Supplementary file 1 [file microorganisms-07-00512-s001.zip › Figure S4.pdf]
